# Do ectomycorrhizal and arbuscular mycorrhizal temperate tree species systematically differ in root order-related fine root morphology and biomass?

**DOI:** 10.3389/fpls.2015.00064

**Published:** 2015-02-11

**Authors:** Petra Kubisch, Dietrich Hertel, Christoph Leuschner

**Affiliations:** Plant Ecology and Ecosystems Research, Albrecht von Haller Institute of Plant Sciences, University of GöttingenGöttingen, Germany

**Keywords:** *Acer*, *Carpinus*, *Fagus*, *Fraxinus*, mixed stand, root tips, specific root area, *Tilia*

## Abstract

While most temperate broad-leaved tree species form ectomycorrhizal (EM) symbioses, a few species have arbuscular mycorrhizas (AM). It is not known whether EM and AM tree species differ systematically with respect to fine root morphology, fine root system size and root functioning. In a species-rich temperate mixed forest, we studied the fine root morphology and biomass of three EM and three AM tree species from the genera *Acer, Carpinus, Fagus, Fraxinus*, and *Tilia* searching for principal differences between EM and AM trees. We further assessed the evidence of convergence or divergence in root traits among the six co-occurring species. Eight fine root morphological and chemical traits were investigated in root segments of the first to fourth root order in three different soil depths and the relative importance of the factors root order, tree species and soil depth for root morphology was determined. Root order was more influential than tree species while soil depth had only a small effect on root morphology All six species showed similar decreases in specific root length and specific root area from the 1st to the 4th root order, while the species patterns differed considerably in root tissue density, root N concentration, and particularly with respect to root tip abundance. Most root morphological traits were not significantly different between EM and AM species (except for specific root area that was larger in AM species), indicating that mycorrhiza type is not a key factor influencing fine root morphology in these species. The order-based root analysis detected species differences more clearly than the simple analysis of bulked fine root mass. Despite convergence in important root traits among AM and EM species, even congeneric species may differ in certain fine root morphological traits. This suggests that, in general, species identity has a larger influence on fine root morphology than mycorrhiza type.

## Introduction

Trees produce large amounts of woody coarse and large roots, but it is the small amount of fine non-woody roots which provide a large surface area and close contact to the soil enabling the absorption of water and nutrients. Conventionally, the most distal short-lived root segments with diameters <2 mm (“fine roots”) are associated with resource acquisition, while the thicker coarse and large roots are considered as being long-lived with transport, storage and anchorage function (Fitter, 1996; Pregitzer, 2002).

Recent root morphological research has shown that the distinction between fine and coarse roots with a fixed diameter threshold of 2 mm is not very useful for categorizing the root system of trees with respect to functionality, metabolic activity, and dynamics (Pregitzer et al., 1997, 2002; Pregitzer, 2002). It appears that certain root properties such as diameter, specific root surface area or tissue N concentration change more or less continuously with increasing distance from the terminal root tip, while anatomical features as cortex thickness, presence of secondary xylem, and the formation of a continuous cork layer as secondary peripheral tissue change more abruptly, perhaps in conjunction with branching events in the fine root system (Pregitzer et al., 2002; Guo et al., 2004). From the analysis of 23 temperate tree species, Guo et al. (2008) concluded that the shift in root function from resource absorption to transport occurs in the third or fourth root order, with branching events in the root system being counted in proximal direction from the terminal tip. Accordingly, root order was found to be a much better predictor of the functioning of a root segment than its diameter.

It is not well known how fine root morphology varies with the taxonomic position and ecology of trees. Differences in phylogenetic relatedness, mycorrhiza type (ectomycorrhizal vs. arbuscular mycorrhizal), growth rate (fast vs. slow), and successional position (early- vs. late-successional) all could possibly influence fine root morphology and fine root system architecture. Theoretically, the variability in fine root morphology among the 1500 or so temperate tree species could be as large as the variation observed in leaf morphology. Alternatively, coexisting tree species from different genera and families could develop convergent patterns of fine root morphology (Withington et al., 2006), at least when growing in the same stand, because a common dominant selective force controls root development. Root order related analysis of 23 Chinese (Guo et al., 2008) and 9 North American temperate tree species (Pregitzer et al., 2002) showed considerable species differences in fine root morphological, anatomical and chemical properties, even though some consistent general trends in branching patterns and anatomy along the fine root branches were detected.

The question of convergence or divergence in root system morphology and functionality is particularly interesting with respect to the distinction between ectomycorrhiza-forming (EM) and arbuscular mycorrhiza-forming (AM) trees. In the overwhelming majority of temperate tree species, the finest rootlets are colonized by ectomycorrhizal (EM) fungi. However, a few AM species are also present, coexisting with EM species in broad-leaved temperate mixed forests. Tree species, which mostly or exclusively form arbuscular mycorrhizas, are present, for example, in the temperate genera *Acer, Fraxinus, Prunus*, and *Liriodendron*. It is generally believed that AM-forming fungi of the phylum Glomeromycota have a positive effect on their host mainly through enhancement of the uptake of inorganic phosphorus, while EM-forming fungi support their host primarily by accessing organic nitrogen compounds (and other nutrient fractions) (George et al., 1995; Read and Perez-Moreno, 2003; Smith et al., 2003; Lang et al., 2011). Because most research on arbuscular mycorrhizas dealt with herbaceous plants, while research on EM primarily focused on trees, a direct functional comparison of these two major types of mycorrhizal association is complicated. We are not aware of a study that systematically searched for principal differences in fine root morphology between temperate EM- and AM-forming trees. Besides species and mycorrhiza type, a third factor with possible influence on fine root morphology is soil depth because soil physics and chemistry are exerting a large influence on root morphogenesis and growth (Wang et al., 2006).

In this study, we examined the variation in fine root morphology and architecture among six co-occurring temperate broad-leaved tree species in a mixed forest, searching for evidence of divergence or convergence in fine root traits under uniform edaphic and climatic conditions. Because root functioning may largely depend on root branching patterns (Pregitzer et al., 1998; Pregitzer, 2002; Guo et al., 2008), we adopted a detailed root order-related analysis of fine root morphology. The six species were from five families (Oleaceae, Betulaceae, Tiliaceae, Fagaceae, and two species from Aceraceae), representing considerable phylogenetic and also functional diversity (three EM and three AM species). We investigated eight root morphological and chemical traits and related the observed trait variation across the six species-sample to the possible influence of root order, tree species, mycorrhiza type and soil depth. We also compared the species in terms of the amount of 1st and 2nd order fine root biomass in the topsoil. Main study goals were (1) to examine whether co-occurring species develop similar patterns of fine root system branching irrespective of phylogenetic relatedness, (2) to search for systematic differences in fine root architecture between EM and AM trees, (3) to compare the species in terms of fine root biomass assigned to root orders, and (4) to assess the advantages of adopting a root order-based analysis over a conventional analysis of bulked fine root material.

## Materials and methods

### Study site

The study site is situated in Hainich National Park in Thuringia, Germany, which harbors old-growth beech forests (*Fagus sylvatica* L.) and relatively species-rich broad-leaved mixed forests on calcareous soil (350 m a.s.l.; 51° 04′ N, 10° 30′ E). Suitable study plots were selected in the “Thiemsburg area” in the north-eastern part of the national park where at least six tree species co-occur either in quasi-random mixture or in small groups consisting of three to six trees of a species. The species considered were those with highest abundance in this mixed forest (Stellario-Carpinetum association, “oak-hornbeam forests”): European beech (*Fagus sylvatica* L.), Small-leaved lime (*Tilia cordata* Mill.), European hornbeam (*Carpinus betulus* L.), European ash (*Fraxinus excelsior* L.), Sycamore maple (*Acer pseudoplatanus* L.) and Norway maple (*Acer platanoides* L.). Three of the six selected species have been found to form AM in Hainich forest (*Acer pseudoplatanus, A. platanoides*, and *Fraxinus excelsior*), the other three (*Carpinus betulus, Fagus sylvatica*, and *Tilia cordata*) EM (Lang et al., 2011). The investigated species are well studied with respect to aboveground morphological and functional properties (Withington et al., 2006; Köcher et al., 2009, 2013; Legner et al., 2013) and also in terms of fine root dynamics and root nitrogen and water uptake capacities (Korn, 2004; Meinen et al., 2009a,b; Jacob et al., 2012; Jacob and Leuschner, 2014; see Table SI 1 in the Supplement). Other forest patches are composed of up to 14 tree species including *Prunus, Ulmus*, and *Quercus* species as well (Meinen et al., 2009b). The majority of trees were about 90–150 years old (Schmidt et al., 2009) and mean canopy height of the dominant trees was 27–32 m with no larger canopy gaps present (average canopy openness 5.7%, Seidel et al., 2012). The herb layer is patchy with an average cover of ~17% in the studied stand (Vockenhuber et al., 2011). The forest was affected by only minor management activities (selective logging) in the past 50 years because part of the stand was used as military training area and all activities ceased in 1997 with the declaration of a national park.

The region has a semi-humid climate [mean annual temperature 7.7°C, mean annual precipitation ~590 mm yr^−1^ (period 1973–2004; Deutscher Wetterdienst, 2005)]. In the study year 2011, a mean annual temperature of 9.5°C and a precipitation of 470 mm yr^−1^ were recorded (data of the nearby weather station Weberstedt/Hainich; Deutscher Wetterdienst, 2009).

The calcareous bedrock (Triassic limestone) is overlain by a base-rich Pleistocene loess layer which led to the development of eutrophic Luvisols (FAO taxonomy 2006) with a profile depth of 60–70 cm as the most widespread soil type in the study region. The soil texture of the mineral soil (0–30 cm) is characterized by high silt (about 74%) and low sand (<5%) contents (Guckland et al., 2009). The soil can dry out strongly in summer and shows partly stagnant properties during spring and winter. Mainly through different foliar nutrient contents, the tree species influence soil chemistry resulting in some variation in topsoil C/N ratio, base saturation and other properties underneath the six tree species (Table 1). *Fagus* patches showed accumulation of organic Ol and Of layers with slightly higher C/N ratio of the mineral topsoil. Topsoil base saturation was somewhat lower under *Fagus* (mean: 89%) than under the other species (range of means: 92–96%) while only minor pH variation was observed (Table 1).

**Table 1:** **Stand and soil properties in the plots of the six species (means ± SE, *n* = 8)**.

**Parameter**	***F. excelsior***	***A. pseudoplatanus***	***A. platanoides***	***C. betulus***	***T. cordata***	***F. sylvatica***
**STAND CHARACTERISTICS**						
Tree height (m)	32.3 ± 1.5	28.6 ± 0.9	23.8 ± 2.0	22.8 ± 1.1	24.2 ± 1.40	26.4 ± 0.7
dbh (cm)	52.2 ± 3.5	58.1 ± 3.2	51.2 ± 3.6	43.4 ± 3.3	46.4 ± 2.3	43.5 ± 2.2
Basal area (m^2^ ha^−1^)	57.1 ± 5.4	47.8 ± 8.8	28.7 ± 2.5	31.6 ± 6.6	50.9 ± 6.0	60.3 ± 7.8
Proportion target species (%)^a^	83.6 ± 3.5	62.7 ± 7.2	77.4 ± 9.9	86.1 ± 8.0	84.8 ± 4.0	90.4 ± 5.7
**SOIL PARAMETERS (MINERAL TOPSOIL)**						
C/N ratio	11.9 ± 0.7	11.6 ± 0.2	12.0 ± 1.0	12.5 ± 0.7	12.0 ± 1.0	12.6 ± 0.4
Base saturation (%)	91.2 ± 4.3	88.8 ± 4.8	87.3 ± 6.8	88.0 ± 0.2	93.4 ± 0.2	78.5 ± 6.8
Water content (%)^b^	37.9 ± 5.5	40.3 ± 1.5	38.8 ± 7.1	37.4 ± 5.0	36.2 ± 3	36.9 ± 2.4
pH (H_2_O)	4.65–6.30	4.77–6.49	4.72–6.96	4.87–6.58	4.81–6.70	4.50–6.12

*The data refer to all trees in a plot of 6 m radius. For pH, the range of values is given*.

a *Of basal area*.

b *May 2012*.

### Study design

Root coring was conducted at 150 cm distance to mature trees of the six target species with diameters at breast height (dbh) of 40–60 cm and presence in the upper canopy layer. We selected either two neighboring trees of the target species and cored between them or conducted the coring in vicinity of one dominant tree of the respective species. This plot selection scheme in the mixed stand minimized possible species effects on soil chemistry (which would have been more pronounced in larger monospecific patches), while it guaranteed that the large majority (typically >80%) of the fine roots belonged to the target species. We sampled eight plots per species (i.e., 48 plots (tree clusters) in total) in a stand area of ~15 ha by randomly selecting trees of suitable species and dimension. Edaphic conditions were sufficiently homogenous to exclude soil-borne effects on fine root morphology, as they have been described by Ostonen et al. (2013). Mean distance between the plots was ~50 m (minimum distance: 6 m) which excludes possible root interactions between neighboring plots in nearly all cases. All stems > 10 cm dbh in a circle of 6 m radius around the root coring location were examined for their species identity, dbh, basal area and tree height (Table 1).

### Soil sampling and fine root extraction

Soil samples for root extraction were collected in June 2011 in the upper 30 cm of the soil in all 48 tree clusters using a steel corer of 35 mm diameter. The extracted soil was separated into the 0–10, 10–20, and 20–30 cm layers and stored in plastic bags at 4°C until final processing was conducted within 3 months. In the laboratory, the soil was gently washed with tap water over a sieve of 0.25 mm mesh width and all fine root branches (diameter <2 mm) of more than 10 mm length picked out with a pair of tweezers, placed under a microscope (6–40 × magnification), separated into live and dead mass and sorted by species. Criteria to distinguish between biomass (live) and necromass (dead) were root turgor, the elasticity of the stele, and the constitution of root stele and periderm (Leuschner et al., 2004; Rewald and Leuschner, 2009; Meinen et al., 2009a,b). Species identification was conducted with a morphological key based on periderm structure and color, root ramification, root tip morphology and the type of mycorrhiza developed which bases on earlier studies in this forest and elsewhere by lab members (Hölscher et al., 2002; Meinen et al., 2009b,c; Jacob et al., 2012). Characteristic branching features and surface properties of the fine root systems of the six species are displayed in pictures compiled in Figure SI 1 in the Supplement, where a brief description of fine root morphology is also given.

For determining the fine root biomass of the six species in the topsoil, the following two-step procedure was applied. After having sorted out the longer fine roots, the amount of finest rootlets <10 mm length was examined in detail under a microscope for half of the samples (4 per species per soil depth). We dispersed the washed sample on filter paper (730 cm^2^) with 36 equal squares marked on it. Six of 36 squares were selected by random and the finest rootlets sorted into living and dead root mass (Van Praag et al., 1988; Hertel and Leuschner, 2002). The biomass and necromass of those six samples was extrapolated to the whole sample and in the following calculated for all samples. Because species identification was hardly possible in this fraction (which represented about 10 percent or less of the overall fine root mass), the species proportions detected in the > 10 mm-samples were applied to this root fraction as well. We considered only the root mass of the tree species (target species and “other species” in a plot) but discarded the root mass of herbaceous species.

### Morphological analysis

All fine root branches of a core were subjected to morphological analysis shortly after collection. This was done separately for the three soil depths. Root segments with diameters >2 mm were cut off. Specific root length (SRL, m g^−1^), specific root area (SRA, cm^2^ g^−1^), root tissue density (RTD, g cm^−3^) and mean root diameter (MD, mm) of the fine root sample were determined for all fine root branches of a sample by placing the roots in Petri dishes filled with purified water for scanning with a flat-bed scanner (EPSON expression 1680, EPSON America Inc.); the scans were analyzed with WinRhizo 2005c software (Régent Instruments Inc., Québec, QC, Canada). The number of root tips was counted in all living root branches under the microscope (6–40 × magnification) and subsequently related to root dry mass. The ectomycorrhizal colonization rate (in %) of the tips was calculated as:

$$(\frac{\text{no}.\text{ of mycorrhizal root tips}}{\text{no}.\text{ of vital root tips}})\;\times \;100$$

In the AM species, the root segments were inspected for colonization by hyphae, but quantitative data were not collected. Subsequently, a representative sub-sample of root strands (1 to maximal 6 per sample) was chosen for a root order-based characterization of traits, and the individual segments of the root samples were assigned to root branching orders according to Strahler's stream ordering system (Pregitzer et al., 2002) and dissected into the orders using a razorblade. For all species except *Fraxinus excelsior*, the root tip(s) plus the consecutive first root segment were counted as first-order segments, as it was not possible to clearly identify the transition between the root tip and the subsequent youngest root segment. In addition, root tips colonized by ectomycorrhizal fungi often formed coralloid clusters that were difficult to split into first and second-order segments. Ash root tips were well visible and were counted as first root order. SRL, SRA, RTD, and MD were separately determined for the root orders 1–4 using the flat-bed scanner and the Win-Rhizo software as described for the fine root bulk samples. In addition, the relative contribution (in percent) of the four root orders to the total biomass, length or surface area of the investigated root strand was determined in order to quantify biomass partitioning in the terminal part of the fine root system. After morphological investigation, the living root-material (entire root branches and separately analyzed fractions in the root orders) and the root necromass were dried at 70°C for 48 h, weighed and ground for analysis of C and N concentrations by gas chromatography (Vario EL, elementar, Hanau, Germany). In the analyses, we distinguish between root order-related data and data relating to the bulk sample (all fine root biomass <2 mm, i.e., all 4 orders combined).

### Data analysis

All data sets were tested for normal distribution using a Shapiro-Wilk test. In most cases, normal distribution was not given and the non-parametric Mann-Whitney *U*-test for pairwise comparisons of means among species, soil depths and root orders was used for all morphological traits. For identifying the principal factors influencing root morphological traits, general linear models (GLM) based on ranks of the independent variables “species,” “soil depth” and “root order” were calculated. Species comparisons (based on means of the 0–30 cm profile) were conducted using a general linear model (GLM) followed by a Scheffé-test. All test statistics were conducted with SAS 9.3 Windows software on a significance level of *p* < 0.05. A Principal Components Analysis was conducted in the software CANOCO (biometris, Wageningen, The Netherlands) to analyze relationships between the five investigated root morphological traits and root orders. Linear and non-linear regressions were calculated with the software Xact7 (Sci Lab, Hamburg, Germany).

## Results

### Species differences in fine root morphology: bulk and root order-related analysis

For comparing the six species, four morphological traits (MD, SRL, SRA, RTD) and root nitrogen concentration were analyzed either for the bulked fine root biomass (all segments <2 mm in diameter pooled) or separately for the root order classes 1–4. In the root order-related analysis, we additionally determined the partitioning of root biomass, root length and surface area to the four studied root orders (expressed in percent of total biomass, length or surface area in the <2 mm class) which allows assessing the relative importance of the four root order classes in the fine root system. The photographs in Figure SI 1 in the Supplement display characteristic fine root strands of the six species.

According to a GLM, all examined root morphological and chemical parameters were strongly dependent on species identity (Table 2). Nevertheless, the species effect was only secondary to the root order effect in all but one trait (RTD). In the bulked samples without separation of root orders, significant species differences existed for SRL and SRA (relatively high in the *Acer* species, intermediate in *C. betulus, F. sylvatica*, and *F. excelsior*, and relatively low in *T. cordata*), RTD (lower in *F. excelsior* than in the other five species), MD (higher in *T. cordata*, and *F. excelsior*, intermediate in *F. sylvatica, A. platanoides*, and *C. betulus*, and lower in *A. pseudoplatanus*) and root N concentration (elevated in *F. excelsior*, intermediate in *C. betulus*, the *Acer* species and *F. sylvatica*, relatively low in *T. cordata*; Table 3).

**Table 2:** **General linear models relating the variables “species,” “soil depth,” “root branching order” and their interactions to the dependent variables root biomass fraction, root surface area fraction, root length fraction, specific root length (SRL), specific root area (SRA), root tissue density (RTD), mean segment diameter (MD) and root N concentration (N) across the sample consisting of six tree species**.

**Dependent variable**	**Model**	**Species**	**Depth**	**Order**	**Species × depth**	**Species × order**	**Depth × order**	**Species × depth × order**
**BIOMASS FRACTION**								
*F*		3.15	4.82	8.33	2.52		2.90	
*p*	0.001	0.01	0.01	0.001	0.01		0.01	
*R*^2^	0.289	0.034	0.021	0.054	0.055		0.038	
**SURFACE AREA FRACTION**								
*F*		6.59	4.22	21.98	3.18		2.18	
*p*	0.001	0.001	0.05	0.001	0.001		0.05	
*R*^2^	0.367	0.064	0.0164	0.128	0.062		0.025	
**LENGTH FRACTION**								
*F*		13.66	21.58	141.06	3.16			
*p*	0.001	0.001	0.001	0.001	0.001			
*R*^2^	0.643	0.074	0.047	0.460	0.034			
**SRL**								
*F*		17.08		252.87				
*p*	0.001	0.001		0.001				
*R*^2^	0.744	0.071		0.628				
**SRA**								
*F*		10.68		157.73				
*p*	0.001	0.001		0.001				
*R*^2^	0.646	0.060		0.535				
**RTD**								
*F*		23.36		7.10		2.24		
*p*	0.001	0.001		0.001		0.01		
*R*^2^	0.393	0.223		0.041		0.060		
**MD**								
*F*		31.24		237.06		2.54		
*p*	0.001	0.001		0.001		0.05		
*R*^2^	0.740	0.124		0.564		0.030		
**N CONCENTRATION**								
*F*		40.22	9.09	111.37	2.14			
*p*	0.001	0.001	0.001	0.001	0.05			
*R*^2^	0.694	0.225	0.020	0.374	0.024			

*Given are the F-value, the significance level (p) and the R^2^ values (only significant factors are presented)*.

**Table 3:** **Five morphological traits of the fine roots (bulk samples; all segments <2 mm in diameter) of the five species in three different soil depths and averaged over the 0–30 cm profile (means ± SE)**.

**Trait**	**Depth (cm)**	***F. excelsior***	***A. pseudoplatanus***	***A. platanoides***	***C. betulus***	***T. cordata***	***F. sylvatica***
RTD (g m^−3^)	0–10	0.341 ± 0.054 bA	0.454 ± 0.038 abA	0.556 ± 0.051 acA	0.493 ± 0.018 aA	0.606 ± 0.058 cA	0.620 ± 0.134 aA
	10–20	0.371 ± 0.033 cA	0.509 ± 0.057 abAB	0.493 ± 0.015 aA	0.514 ± 0.031 abA	0.563 ± 0.067 bA	0.487 ± 0.041 aA
	20–30	0.378 ± 0.047 cA	0.540 ± 0.031 abB	0.462 ± 0.099 abcA	0.460 ± 0.026 aA	0.478 ± 0.053 abcA	0.536 ± 0.037 bA
	Profile average	0.340 ± 0.042 α	0.491 ± 0.026 α	0.576 ± 0.094 α	0.488 ± 0.013 α	0.525 ± 0.036 α	0.521 ± 0.043 α
MD (mm)	0–10	0.482 ± 0.029 cA	0.317 ± 0.006 bA	0.436 ± 0.070 acA	0.374 ± 0.023 aA	0.468 ± 0.056 acA	0.411 ± 0.103 abcA
	10–20	0.524 ± 0.054 cA	0.318 ± 0.026 bA	0.394 ± 0.019 aA	0.363 ± 0.021 abA	0.567 ± 0.073 cA	0.460 ± 0.053 acA
	20–30	0.587 ± 0.070 bA	0.363 ± 0.034 aA	0.476 ± 0.105 abA	0.511 ± 0.082 abA	0.652 ± 0.093 bA	0.459 ± 0.087 aA
	Profile average	0.531 ± 0.030 αβ	0.333 ± 0.015 α	0.416 ± 0.027 αβ	0.416 ± 0.028 αβ	0.562 ± 0.045 β	0.459 ± 0.067 αβ
SRA (cm^2^ g^−1^)	0–10	183.0 ± 13.3 cA	227.1 ± 8.6 bA	150.3 ± 8.6 aA	180.4 ± 28.0 abA	118.0 ± 12.2 cA	191.1 ± 62.6 abA
	10–20	154.2 ± 20.2 abA	245.5 ± 76.1 abcAB	176.1 ± 21.0 aA	160.7 ± 0.9 abA	103.7 ± 35.2 cA	125.8 ± 17.2 bcA
	20–30	159.5 ± 20.2 aA	163.3 ± 19.4 aB	233.2 ± 72.4 abA	126.2 ± 22.6 abA	107.4 ± 18.4 bA	121.6 ± 19.5 abA
	Profile average	165.1 ± 15.26 αβ	183.6 ± 20.0 α	145.4 ± 13.2 αβ	142.9 ± 19.0 αβ	98.7 ± 15.3 β	119.5 ± 19.1 αβ
SRL (m g^−1^)	0–10	13.536 ± 1.291 aA	28.038 ± 2.653 bA	15.313 ± 1.922 aA	21.994 ± 5.411 abA	10.911 ± 2.027 aA	29.240 ± 12.629 abA
	10–20	10.921 ± 1.819 bcA	34.769 ± 13.013 aAB	18.612 ± 3.290 abcA	17.615 ± 3.154 abA	7.455 ± 3.408 cA	12.552 ± 2.656 bA
	20–30	10.894 ± 2.130 bcA	19.472 ± 3.310 aB	22.831 ± 7.610 abcA	11.587 ± 3.509 abcA	7.436 ± 1.943 cA	12.797 ± 2.843 abA
	Profile average	11.717 ± 1.367 αβ	22.394 ± 3.151 α	13.939 ± 1.396 αβ	14.967 ± 3.193 αβ	7.614 ± 1.698 β	12.292 ± 2.935 αβ
N (mg g^−1^)	0–10	14.267 ± 0.682 cA	11.635 ± 0.551 bA	9.902 ± 0.621 aA	12.196 ± 1.122 abcA	9.953 ± 0.805 abA	10.946 ± 0.943 abA
	10–20	13.440 ± 0.681 cA	9.519 ± 0.522 aB	8.892 ± 0.480 aA	11.362 ± 0.837 bcAB	7.923 ± 0.349 dB	10.315 ± 0.853 abA
	20–30	13.344 ± 0.600 bA	10.076 ± 0.841 aAB	10.469 ± 0.982 aA	9.191 ± 0.817 aB	8.988 ± 0.322 aA	9.102 ± 1.125 aA
	Profile average	13.601 ± 0.434 β	9.442 ± 0.813 α	9.221 ± 0.229 α	11.110 ± 1.005 αβ	9.279 ± 3.030 α	9.636 ± 0.708 α

*RTD, root tissue density; MD, mean diameter in <2 mm class; SRA, specific root area; SRL, specific root length; N, root N concentration. Differences between the species in a soil depth are marked by different lower case letters, differences between the soil depths by capital letters. Species differences in the profile average are indicated by different greek letters*.

The number of root tips per fine root mass was relatively low in *F. excelsior* and *T. cordata*, intermediate in *A. platanoides, C. betulus*, and *F. sylvatica*, and highest in *A. pseudoplatanus*. Correspondingly, the number of tips per soil volume was particularly large in *A. pseudoplatanus* and *F. sylvatica* and low in *F. excelsior, T. cordata* and *A. platanoides* (Table 4).

**Table 4:** **Root tips per biomass or soil volume, proportion of root tips colonized by EM fungi, tips per square meter soil and cumulative length of 1st-order root segments per liter soil volume for the six species in the three horizons and the entire profile (0–30 cm)**.

**Species**	**Soil depth (cm)**	**Tips per FR biomass (n g^−1^)**	**Tips per soil volume (n L^−1^)**	**Tips per square meter (n m^−2^)**	**Proportion infected (%)**	**Cumul. length of 1st order roots per soil volume (m L^−1^)**
*F. excelsior*	0–10	1807 cA	2092 bA	209151	–	8.13 ± 1.24 dcA
	10–20	1328 bA	1227 aAB	107352	–	5.10 ± 1.15 aAB
	20–30	1654 bcA	878 aB	87799	–	2.89 ± 0.73 abB
Profile average	0–30	1466 γ	1169 α	331894 αχ	–	5.38 ± 0.71 αβ
*A. pseudoplatanus*	0–10	11297 bA	7559 cA	755902	–	11.22 ± 0.96 cA
	10–20	9031 aAB	3899 cB	389863	–	6.32 ± 1.30 aB
	20–30	7155 aB	2861 bB	286061	–	4.38 ± 1.09 aB
Profile average	0–30	8557 β	4084 β	1439768 βδ	–	7.31 ± 0.56 β
*A. platanoides*	0–10	4948 aA	2349 aA	234907	–	4.63 ± 1.31 abAB
	10–20	5684 aA	2063 aA	206294	–	4.49 ± 0.98 aA
	20–30	8662 abA	720 aB	72047	–	1.62 ± 0.81 bB
Profile average	0–30	4148 α	1178 α	509526 αχδ	–	2.99 ± 0.71 α
*C. betulus*	0–10	8832 abA	4541 aA	454148	85.8 ± 4	5.50 ± 1.24 adAB
	10–20	5034 aA	3799 bA	379855	89.1 ± 3	6.62 ± 0.98 bA
	20–30	3416 abcA	1176 bB	117579	98.1 ± 1	2.93 ± 0.81 abB
Profile average	0–30	4578 α	2110 γ	956849 βχδ	90.0 ± 2	4.77 ± 0.72 αβ
*T. cordata*	0–10	4769 aB	1738 abA	173775	74.8 ± 8	2.25 ± 0.85 bA
	10–20	3789 bA	2773 abA	277295	76.5 ± 8	3.15 ± 1.37 acA
	20–30	1909 cA	1184 abA	118392	77.1 ± 5	3.37 ± 0.39 abA
Profile average	0–30	2888 γδ	1354 αγ	607816 χδ	76.0 ± 4	2.14 ± 0.63 α
*F. sylvatica*	0–10	11427 abcA	3915 abA	395314	88.8 ± 6	4.65 ± 1.74 abdAB
	10–20	4604 abA	5410 bA	540977	86.3 ± 4	8.40 ± 1.34 cbA
	20–30	5323 aA	3686 bA	368591	91.7 ± 4	5.06 ± 1.25 aB
Profile average	0–30	5129 αβδ	3251 αβγ	1197948 δ	89.0 ± 2	5.30 ± 1.32 αβ

*Significant differences between species per soil depth are indicated by different lower case letters, differences for a species between soil depths by capital letters, differences between profile averages by greek letters*.

**Figure 1:**
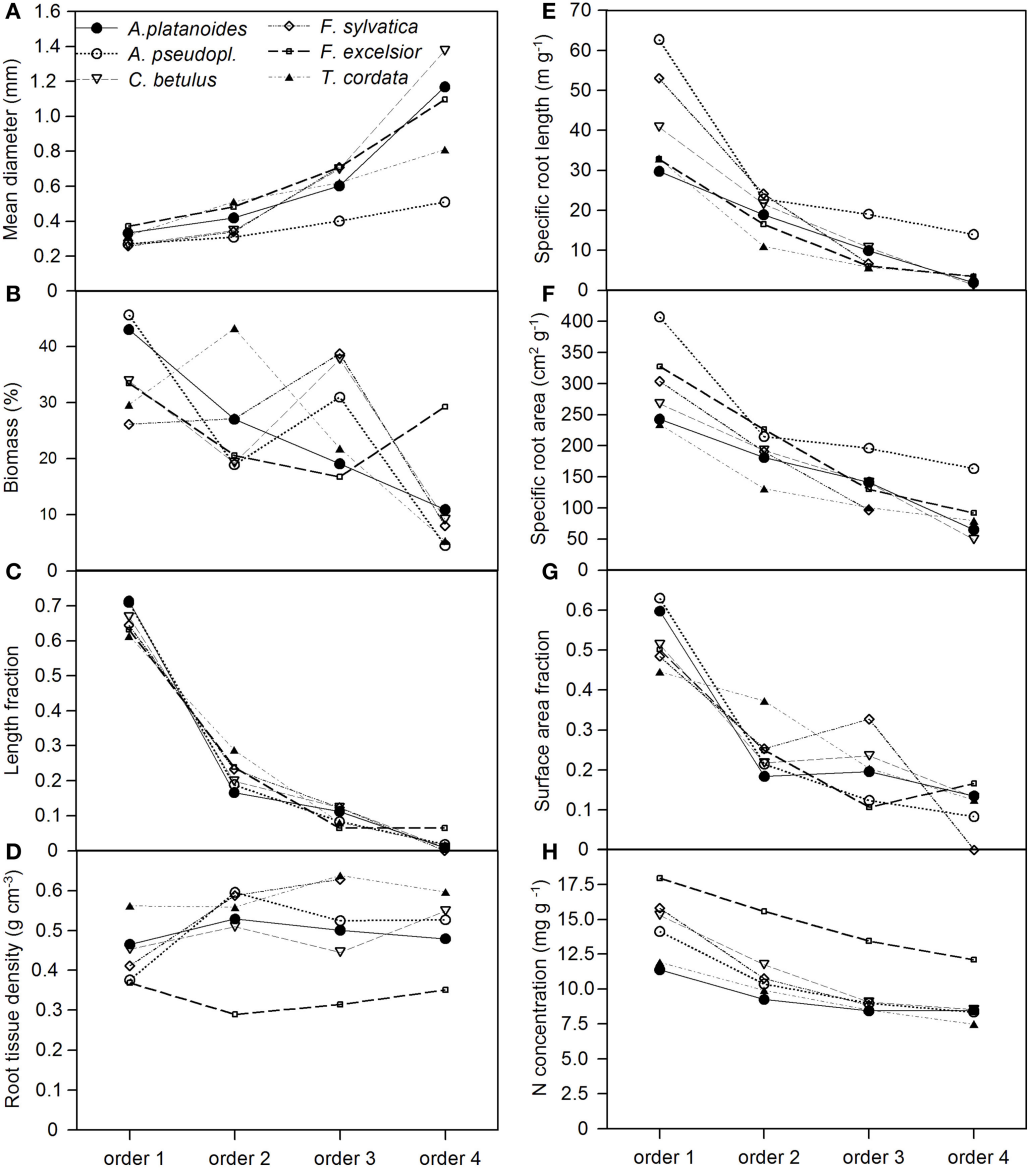
**Change in eight root morphological or chemical parameters **(A–H)** along fine root strands from the first to the fourth root order in the six tree species (given are means of 8 replicate plots that were cored; each sample consisted of 1–6 roots that were averaged)**. All root strands had a maximum diameter of 2 mm. The data refer to the 0–10 cm layer.

The two congeners *A. pseudoplatanus* and *A. platanoides* had a remarkably different fine root morphology, in particular with respect to SRL and SRA (Figures 1E,F), even though they appeared to be morphologically similar under the microscope (Figure SI 1). The first-order rootlets of *A. pseudoplatanus* had a significantly higher SRL and SRA with a tendency for higher N concentration than those of *A. platanoides* in all three soil depths. *A. pseudoplatanus* also produced thinner 4th-order root segments with higher SRA than its congener (Figures 1A,E,F and Figure SI 1 in the Supplement). Further, *A. pseudoplatanus* forms significantly more fine root tips (per root mass and per soil volume) than *A. platanoides* (Table 4).

When comparing the three EM and three AM species, significant differences were only detected for one of the eight traits; SRA was larger in the AM than the EM species (Table 5). We did not get hints on systematic differences in root tip frequency between AM and EM species (Tables 4, 5) as it might be expected from the largely different morphology of the two mycorrhiza types. Downward in the soil profile (from 0–10 to 20–30 cm), the AM species showed significant decreases in the number of fine root tips per soil volume and the cumulative length of first-order rootlets (which contain the tips) per volume; in contrast, no such trend was visible in the EM species (Table 4). However, AM and EM species did not differ significantly with respect to tip numbers per soil volume.

**Table 5:** **Means ± SE of seven root morphological or chemical traits for the each three AM and EM species (data averaged over the 0–30 cm profile)**.

**Mycorrhiza type**	**MD (mm)**	**SRA (cm^2^ g^−1^)**	**SRL (m g^−1^)**	**RTD (g cm^−3^)**	**N (mg g^−1^)**	**Tips per mass (g^−1^)**	**Tips per volume (L^−1^)**
AM	0.42 ± 0.02	164.64 ± 9.75	16.20 ± 1.56	0.47 ± 0.04	11.77 ± 0.53	4865 ± 821	2186 ± 350
EM	0.48 ± 0.03	120.34 ± 10.54	11.62 ± 1.61	0.51 ± 0.02	10.01 ± 0.46	4198 ± 702	2238 ± 394
p	0.33	0.04^*^	0.33	0.50	0.50	0.50	0.68

Given is the p-value of a comparison of the means (Mann-Whitney U-test) and the significance level (

* *p <0.05)*.

In general, the influence of soil depth on root morphology was relatively small. Only in a few cases, we observed significant directional change in fine root morphological traits from the 0–10 to the 20–30 cm layer. Notable is the increase in RTD with soil depth in *A. pseudoplatanus* and the decrease in root N concentration in *C. betulus* (Table 3).

Root order was the key factor influencing fine root traits (Table 2). According to the PCA, the morphological traits MD, SRA, and SRL showed the closest association with root order, which was located on the first PCA axis. In contrast, the association with order was weaker for the anatomical and chemical parameters N concentration and RTD (Table 6). All species showed a similar increase in root diameter and a decrease in SRA, SRL, and N concentration from the first to the fourth order, while the RTD pattern along the root was more variable among the species. *F. excelsior* differed from the other species by particularly low RTD and high N concentrations in all root orders (Figures 1D,H). The root order-based analysis revealed that the species differences were often pronounced in one order but negligible in others (as visible in MD and RTD; see Figure 1).

**Table 6:** **Principal components for the relatedness of eight root morphological and chemical traits and root order (order 1–3) with the axes 1–4 of a PCA covering all six species (in brackets: cumulative fit values *R*^2^)**.

**Variables**	**Axis1**	**Axis2**	**Axis3**	**Axis4**
EV	0.738	0.133	0.073	0.039
Root branching order	**−0.944 (0.890)**	0.164 (0.917)	−0.044 (0.919)	0.206 (0.962)
SRL (m g^−1^)	**0.922 (0.850)**	−0.207 (0.894)	−0.118 (0.908)	0.284 (0.988)
SRA (cm^2^ g^−1^)	**0.966 (0.933)**	0 (0.933)	−0.041 (0.935)	0.217 (0.982)
RTD (g cm^−3^)	**−0.628 (0.394)**	**−0.697 (0.880)**	0.334 (0.992)	0.090 (1.000)
N concentration (mg g^−1^)	**0.777 (0.604)**	0.310 (0.700)	0.545 (0.997)	−0.015 (0.997)
Mean diameter (mm)	**−0.870 (0.756)**	0.383 (0.903)	0.123 (0.918)	0.241 (0.976)

*The eigenvalues of the axes are given in the second row. The closest correlations of the components with the respective axis are given in bold print*.

### Species differences in the abundance and distribution of fine root biomass

Total fine root biomass in the 0–30 cm profile (bulked samples) differed up to twofold among the six species with highest plot mean in *F. sylvatica* (301 g m^−2^) and lowest in *A. platanoides* (142 g m^−2^, difference significant at p <0.05; Figure SI 2 in the Supplement). In the profile totals, ~89–95% of root biomass was contributed by the target species and the remainder (<25 g m^−2^ in 0–30 cm) by other woody species that grew in the neighborhood. Part of the species differences in fine root biomass seem to be caused by differences in the species' aboveground presence in the plots as indicated by the variable basal areas of the species in the plots (28.7–60.3 m^2^ ha^−1^, Table 1). However, the species may also differ inherently in their fine root biomass in the upper soil as indicated by large species differences in the fine root biomass/basal area ratio of the plots (range: 50–124 g fine root biomass per m^2^ basal area in the six species; data not shown).

Fine roots of *F. sylvatica* and *T. cordata* seemed to prefer the 10–20 cm layer (47 and 43% of the biomass profile total) over the top layer (0–10 cm) in the respective plots, while the other species showed similar fine root densities at 0–10, 10–20, and 20–30 cm depth (or a reduced density at 20–30 cm, Figure SI 2). Correspondingly, GLMs showed that soil depth had a smaller influence on fine root biomass variation across the study plots than species identity (Table 2).

### Species differences in the abundance of 1st- and 2nd-order fine root biomass

**Figure 2:**
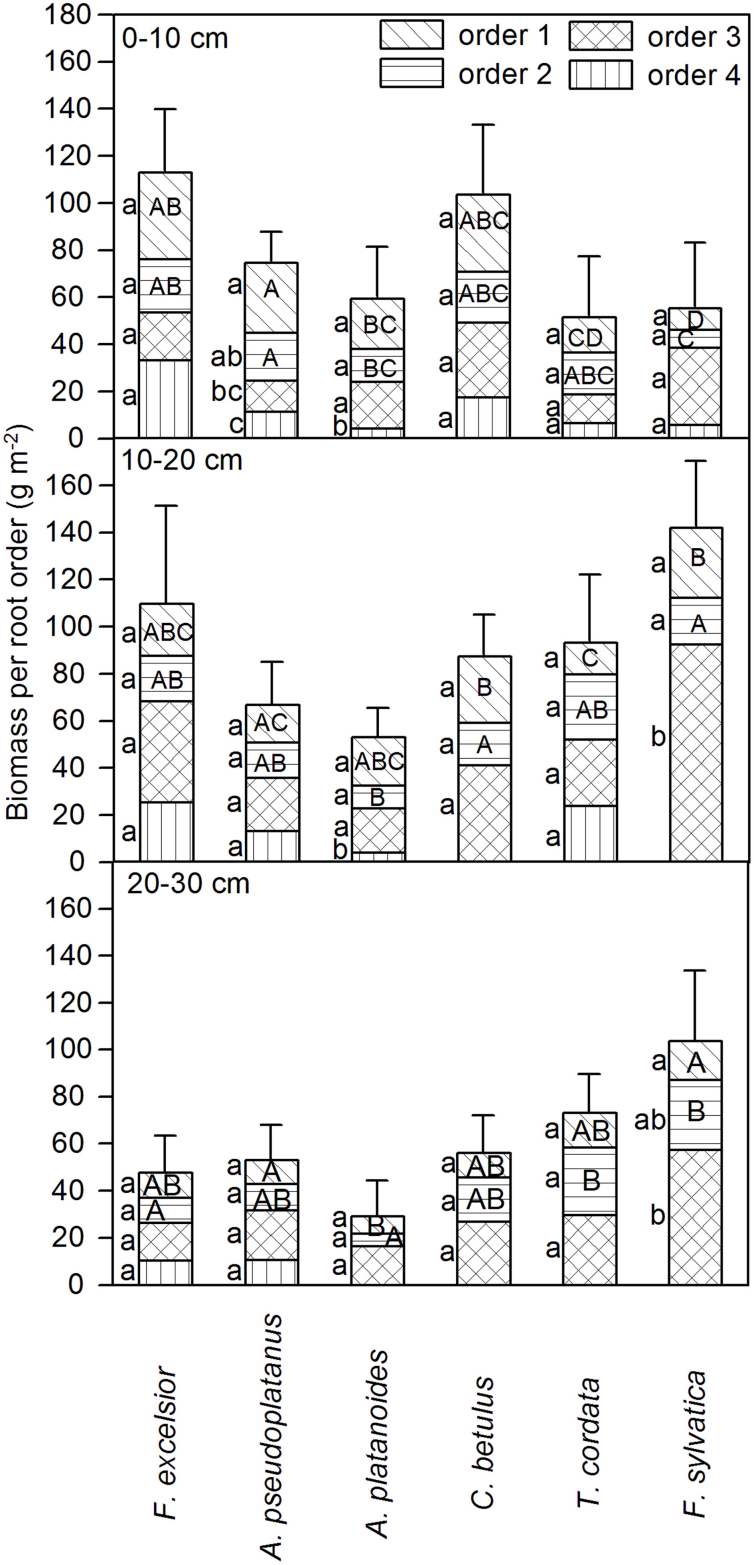
**Fine root biomass assigned to the root orders 1–4 (uppermost to lowermost sections of bars) in three soil depths (0–10, 10–20, and 20–30 cm, in g m^−2^ 10 cm depth^−1^) for the six tree species (means ± SE)**. Different small letters mark significant differences between root orders for a species, different capital letters significant differences in a given order between the species (only 1st and 2nd order); Mann-Whitney *U*-test; *p* < 0.05.

A comparison of the six species with respect to the abundance of root biomass assignable to the 1st or 2nd root orders revealed species differences in root system structure that would not have been detected by a comparison of bulked fine root biomass totals (Figure 2). *F. sylvatica* and *T. cordata* had a significantly smaller 1st- and 2nd-order root biomass in the 0–10 cm layer than the other species. Due to the relatively small biomass proportion of the two species in these root orders, beech and lime differed from the other species more in 1st- and 2nd-order root biomass than in total fine root biomass. *A. pseudoplatanus* had significantly more 1st- and 2nd-order root biomass in the 0–10 cm layer than its congener *A. platanoides*. Highest values in this layer were reached by *F. excelsior*.

In 10–20 cm depth, most species had higher amounts of 3rd- and 4th-order roots than in the top layer. 1st- and 2nd-order fine root biomass decreased toward the 20–30 cm layer and the degree of root branching decreased as well. As a consequence, four of the six species possessed only three root orders in this soil layer until roots exceeded 2 mm in diameter (Figure 2); thicker segments were cut off prior to analysis and thus were not investigated here.

## Discussion

### Interspecific variation in fine root morphology and branching patterns

Our root order-based analysis produced evidence for convergence in important fine root morphological traits and branching patterns across the six investigated species, even though most taxa were not closely related to each other and had a largely different ecology. In particular the functionally important traits SRA, length fraction and surface area fraction, which determine the development of root surface area in the most uptake-active 1st- and 2nd-order root segments, showed coherent patterns of change from the first to the fourth root order in all species. Further, the relative variation among the species was smaller than in several important aboveground traits such as leaf size, the size difference between sun and shade leaves, or mean xylem vessel diameter (Köcher et al., 2013; Legner et al., 2013).

On the other hand, several other root traits differed largely between the species. Most variable was root tip frequency (the number of tips per fine root mass) with ~6 fold difference between the species (smallest in *F. excelsior*, largest in *A. pseudoplatanus*) matching results of Ostonen et al. (2007) in three boreal tree species. More relevant for nutrient uptake capacity may be the number of tips per soil volume which still differed about 3.5 fold among the co-occurring species. This variability is also visible in species differences in the cumulative length of 1st-order root segments per soil volume: species means ranged from 2.14 to 7.31 m L^−1^ soil; a substantial part of the first order segment is contributed by the root tip itself. The length of the hyphal net per soil volume is certainly another, probably even more important, morphological factor influencing uptake capacity. Unfortunately, we do not have information on this variable.

A relatively high species variation existed also in the pattern as to how root tissue density and root N concentration changed from the first to the fourth root order. *F. excelsior* differed substantially from the other species with lower RTD and higher N in particular in the second to fourth orders. Thus, apparent convergence in several root traits can go along with markedly diverging patterns in other properties. We reached at similar conclusions when the different soil layers were analyzed separately or the pooled samples of all soil layers were examined. In fact, soil depth exerted only a minor influence on fine root morphology and branching patterns of these six species.

When interpreting the findings from Hainich forest, it is important to recognize that convergence in root traits was detected for the modes of C and N allocation within the fine root system, i.e., the trees' strategy to use plant resources for generating nutrient and water capturing surfaces. We speculate that a relatively compact soil (clay content: 20–30%) with relatively high bulk density (~1.2 g cm^−3^ in the topsoil) and temporal desiccation in dry summer periods may represent conditions favoring convergence in the root traits examined. All species must face similar physical root growth constraints and a comparable carbon-investment-to-nutrient-return ratio of roots exploring the soil. The much larger species variation in root tip numbers than in branching patterns indicates that this trait must be more under genotypic control than others. Observed species differences in root mass- and surface area-specific nutrient uptake capacity (e.g., Jacob and Leuschner, 2014) might, in part, be a consequence of differences in root tip numbers, but such dependence has not yet been examined. Alternatively, differences in hyphal length and activity, and in root activity per root surface area, may also be influential factors.

The partly deviating fine root properties of *F. excelsior* (relatively thick, N-rich 1st- and 2nd-order roots with low tissue density and only few root tips), which have already been noted in earlier studies (Meinen et al., 2009a; Jacob et al., 2012), could relate to the ecology of this species. *F. excelsior* differs in important functional traits from the other investigated species, notably in its relatively high growth rate as an early- to mid-successional species, its ring-porous xylem with large vessels in the stem, and a relatively high N demand (Ellenberg and Leuschner, 2010; Dobrowolska et al., 2011). *F. excelsior* further deviated from the other species by a particularly low root biomass: necromass ratio which may point to species differences in fine root mortality in this mixed stand. One might assume that ash as a species with preference of base-rich fertile soils does require lower root tip numbers than other species, but all six species of our study grew on similar soil.

Species differences in fine root properties were also notable between the two closely related *Acer* species (particularly high SRL and SRA in 1st-order roots of *A. pseudoplatanus*) which is in agreement with the results of Hölscher et al. (2002).

### Root morphological differences between EM and AM tree species

To our surprise, we found significant differences between the three EM and three AM species in only one of the seven root morphological, chemical or branching-related traits. In both groups, considerable among-species variation existed for the variables tip number per root mass and tip number per soil volume. Despite the contrasting modes of interaction between root and fungus in the two mycorrhiza types, the EM species (*C. betulus, T. cordata, F. sylvatica*) on average did not form more root tips per root mass or soil volume than the AM species (*Acer* spp. and *F. excelsior*), where the fungus infects larger sections of the root than in EM trees. In our EM species, we observed insertion of hyphae mainly in the tip with the Hartig net but also in the directly adjacent parts of the 1st and 2nd root orders. While nearly all tips were colonized by fungi in the three EM species (~96% according to the study of Lang et al., 2011 in Hainich forest), only about 19% of the roots of *Fraxinus* and *Acer* were found to be infected by AM fungi (Lang et al., 2011). Largely different between the two groups was also the diversity of colonizing fungi in this mixed forest (75, 68, and 43 EM fungal species in *Fagus, Tilia*, and *Carpinus*, and 7 different taxa of glomeromycota in the AM species according to ITS sequencing, Lang et al., 2011). It appears that, at least in the studied six species, the type of mycorrhiza is not an important determinant of fine root branching patterns and morphology despite the contrasting patterns of symbiotic interaction. This is somewhat surprising because it is well recognized that colonizing EM fungi have major effects on root morphology and architecture by inducing the formation of short lateral roots and root tips that become swollen with a coralloid or “Christmas-tree” like structure (Smith and Read, 1997). Infection by AM-forming fungi appears to have more subtle effects on root morphology and architecture with changes observed in branching patterns and in the length of 2nd- and 3rd-order root segments (Hetrick, 1991; Hooker et al., 1992). Thus, infections either by glomeromycota (AM) or basidio- or ascomycetes (EM) both tend to alter fine root morphology, but the morphogenetic effect has not yet been compared for AM and EM trees in a shared soil volume. We also found no clear hints for an important influence of mycorrhiza type on root functioning, because aboveground productivity was not systematically different between the AM and EM trees in Hainich forest, and neither foliar nor fine root N concentration showed clear differences between EM and AM species in this forest (Jacob et al., 2010).

At least in the fertile soils of Hainich forest, other factors such as species differences in standing fine root biomass, in fine root turnover, and in local nutrient availability as resulting from tree species effects on soil chemistry (Rothe and Binkley, 2001; Guckland et al., 2009) may be more relevant for root functioning than the type of mycorrhiza. The sheer number of root tips also does not seem to be a relevant factor for tree nutrition in this forest, because *A. pseudoplatanus* with highest fine root tip numbers per root mass and soil volume among the six species did not possess higher fine root and foliar N concentrations and was not more productive than the other species.

### The importance of 1st- and 2nd-order root segments

The root order-related analysis of fine root biomass showed that only a half to a third of the conventionally sampled fine root biomass (<2 mm in diameter) referred to 1st- and 2nd-order segments in our study and that this fraction was more variable among the six species than bulk fine root biomass. We also found that the relative proportion of these two root fractions is highest in the topsoil (0–10 cm), while 3rd- and 4th-order segments are more important lower down in the profile where the supply of nitrogen (and other nutrients) is lower and small-diameter roots may primarily have transport functions. Lower root physiological activity deeper in the soil is also suggested by an increasing root C/N ratio with increasing soil depth in our soil profiles (data not shown); this matches results of Gaul et al. (2009) from spruce forest soils.

Our data on order-specific fine root biomass per ground area of mature trees can be compared with only very few other studies (e.g., Guo et al., 2004; Wang et al., 2006; Sun et al., 2011). These studies are, however, only partly equivalent to our study because they refer to immature stands, sampled only the topsoil (0–10 or 20 cm) and compared only two species with inclusion of conifers. Nevertheless, it appears that different tree species may differ considerably with respect to the proportion of 1st- and 2nd-order roots in fine root biomass. More comparative studies in other forest types are needed for quantifying this root fraction and examining the link to tree resource uptake and productivity.

## Conclusions

Comparative fine root system analysis in the Hainich mixed forest, either by examining bulked fine root samples (<2 mm in diameter) or through detailed analysis of root orders, revealed that the more precise, but highly labor-intensive, order-based analysis detected several species differences that would have been overlooked in the more rapid analysis of bulked samples. However, species differences in the important traits SRA, root N concentration and MD were also reflected in the bulk analysis. Thus, for many purposes, it may be sufficient to analyze bulked root material (e.g., in the diameter class <1 or <2 mm) for characterizing morphological differences between, and similarities among, temperate tree species. Nevertheless, studies in additional tree species have to show, whether the detected convergent patterns in SRA and in the length and surface area fractions along fine root strands are indeed more or less similar among different temperate tree species.

The comparison of AM and EM tree species revealed no systematic fine root morphological differences between the two mycorrhiza types except for SRA. We suggest searching more systematically for different structural and functional consequences of the formation of either AM or EM symbioses in temperate tree species. Our approach of investigating arbuscular and ectomycorrhizal species of the same plant life form in a mixed stand may shed new light on the old discussion about principal functional differences between these two types of plant-fungus interaction.

### Conflict of interest statement

The authors declare that the research was conducted in the absence of any commercial or financial relationships that could be construed as a potential conflict of interest.
