# Supplementary material for: Do ectomycorrhizal and arbuscular mycorrhizal temperate tree species systematically differ in root order-related fine root morphology and biomass?
Source: Front Plant Sci. 2015 Feb 11;6:64. doi: 10.3389/fpls.2015.00064 (PMC4324066; doi:10.3389/fpls.2015.00064)
Supplement: Supplementary file 1 [file Table1.PDF]

Table SI 1. Some morphological and functional traits of the six studied species according to different sources.

	Fraxinus excelsior	*Acer pseudo-platanus*	*Acer platanoides*	*Carpinus betulus*	*Tilia cordata*	*Fagus sylvatica*
Position in succession [a]	early-mid	mid/late	mid/late	mid/late	mid/late	late
Mycorrhiza type [b]	AM	AM	AM	EM	EM	EM
Wood density (g cm^{-3}) [c]	0.59	0.59	0.62	0.67	0.43	0.65
Sun leaf SLA (cm^2 g^{-1}) [d]	80.1	79.2	n.d.	100.7	102.5	86.9
Below-canopy shade intensity [e]	moderate	high	high	very high	high	very high
Xylem anatomy	ring	diffuse	diffuse	diffuse	diffuse	diffuse
Drought sensitivity [f]	low	moderate-high	moderate	moderate-low	moderate-low	high

[a] according to Ellenberg & Leuschner (2010) and other sources
[b] according to root studies in Hainich forest by Lang et al. (2011)
[c] after different sources
[d] after Legner et al. (2013) (mature trees in Hainich forest)
[e] according to Ellenberg & Leuschner (2010)
[f] after Köcher et al. (2009) and Hölscher et al. (2002)
