# Supplementary material for: Do ectomycorrhizal and arbuscular mycorrhizal temperate tree species systematically differ in root order-related fine root morphology and biomass?
Source: Front Plant Sci. 2015 Feb 11;6:64. doi: 10.3389/fpls.2015.00064 (PMC4324066; doi:10.3389/fpls.2015.00064)
Supplement: Supplementary file 2 [file Image1.PDF]

Figure SI1. Exemplary pictures of morphological characteristics of fine roots of the six broad-leaved tree species investigated in this study. *Acer platanoides* (A-C): colour of the thicker main axes ochre to dark brown; root tips of very small diameter, typically colonized with arbuscular mycorrhizal (AM) fungi (not ectomycorrizal EM), often very hairy, not transparent, connection to the consecutive fine root branch a bit constricted; surface of the thicker main axes with rough longitudinal net structure of older periderm parts, younger periderm parts smooth with shining fine cross-striped structure; second and higher order branches very irregularly ramified and tortuous. *Acer pseudoplatanus* (D-F): colour of the thicker main axes beige to dark brown; root tips of very small diameter, typically colonized with AM fungi (not EM), not very hairy, often transparent, connection to the consecutive fine root branch clearly constricted; surface of the thicker main axes with rough dark longitudinal net structure of older periderm parts, younger periderm parts smooth with shining fine cross-striped structure; second and higher order branches very irregularly ramified and tortuous. *Carpinus betulus* (G-I): colour of the thicker main axes dark red to dark brown; root tips typically colonized with EM; surface of the thicker main axes with very regular narrow longitudinal furrows, no clear differentiation of older from younger periderm parts; second and higher order branches irregularly ramified and often straight-line structured. *Fagus sylvatica* (K-M): colour of the thicker main axes red to reddish brown; root tips typically colonized with EM; surface of the thicker main axes with narrow or wide longitudinal furrows, no clear differentiation of older from younger periderm parts; second and higher order branches irregularly ramified and often tortuous. *Fraxinus excelsior* (N-P): colour of the thicker (and also thinner!) main axes beige or light brown to grey brown; root tips typically colonized with AM fungi (not EM); root tips and all other branch orders dense hairy; surface of the thicker main axes as of thinner axes with little or unclear surface structure, mostly no older periderm parts visible; second and higher order branches very regularly ramified and not very tortuous. *Tilia cordata* (Q-S): colour of the thicker main axes beige to amber-coloured or dark brown; root tips typically colonized with EM; surface of the thicker main axes with rough dark longitudinal slabby net structure of older periderm parts, younger periderm parts smooth with shining fine cross-striped structure; second and higher order branches very irregularly ramified and medium tortuous.

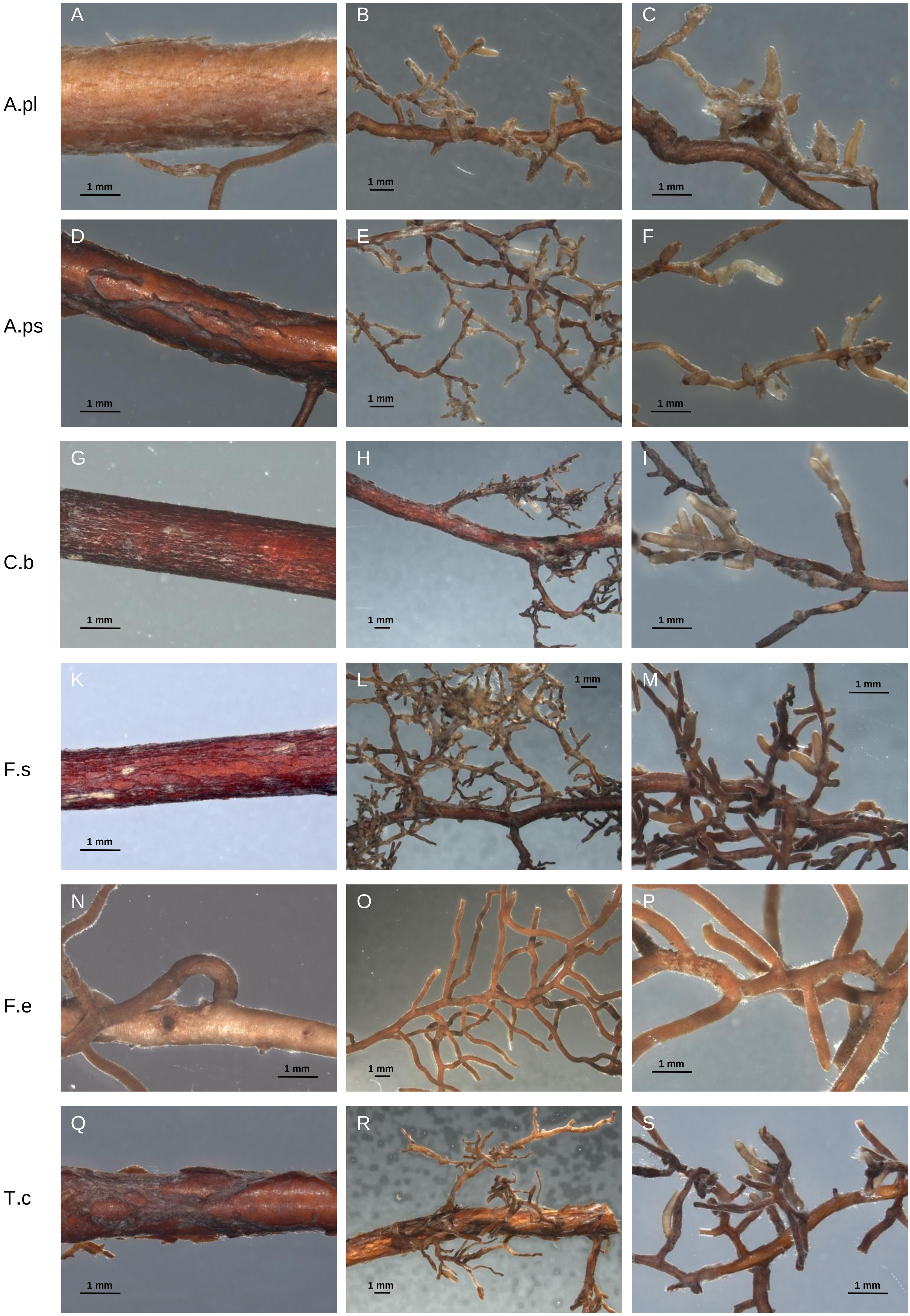
A.pl
A
B
C
A.ps
D
E
F
C.b
G
H
I
F.s
K
L
M
F.e
N
O
P
T.c
Q
R
S
1 mm
