# Supplementary material for: Do ectomycorrhizal and arbuscular mycorrhizal temperate tree species systematically differ in root order-related fine root morphology and biomass?
Source: Front Plant Sci. 2015 Feb 11;6:64. doi: 10.3389/fpls.2015.00064 (PMC4324066; doi:10.3389/fpls.2015.00064)

Figure SI 2. Fine root biomass (A) and necromass (B) in three soil layers in the plots of the six species (n = 8 plots; respective left bar: target species, small right bar: other tree species present in 0-30 cm depth). Given are means ± SE. Significant differences between the species (profile total) are indicated by different letters (Mann-Whitney U test, $p < 0.05$).

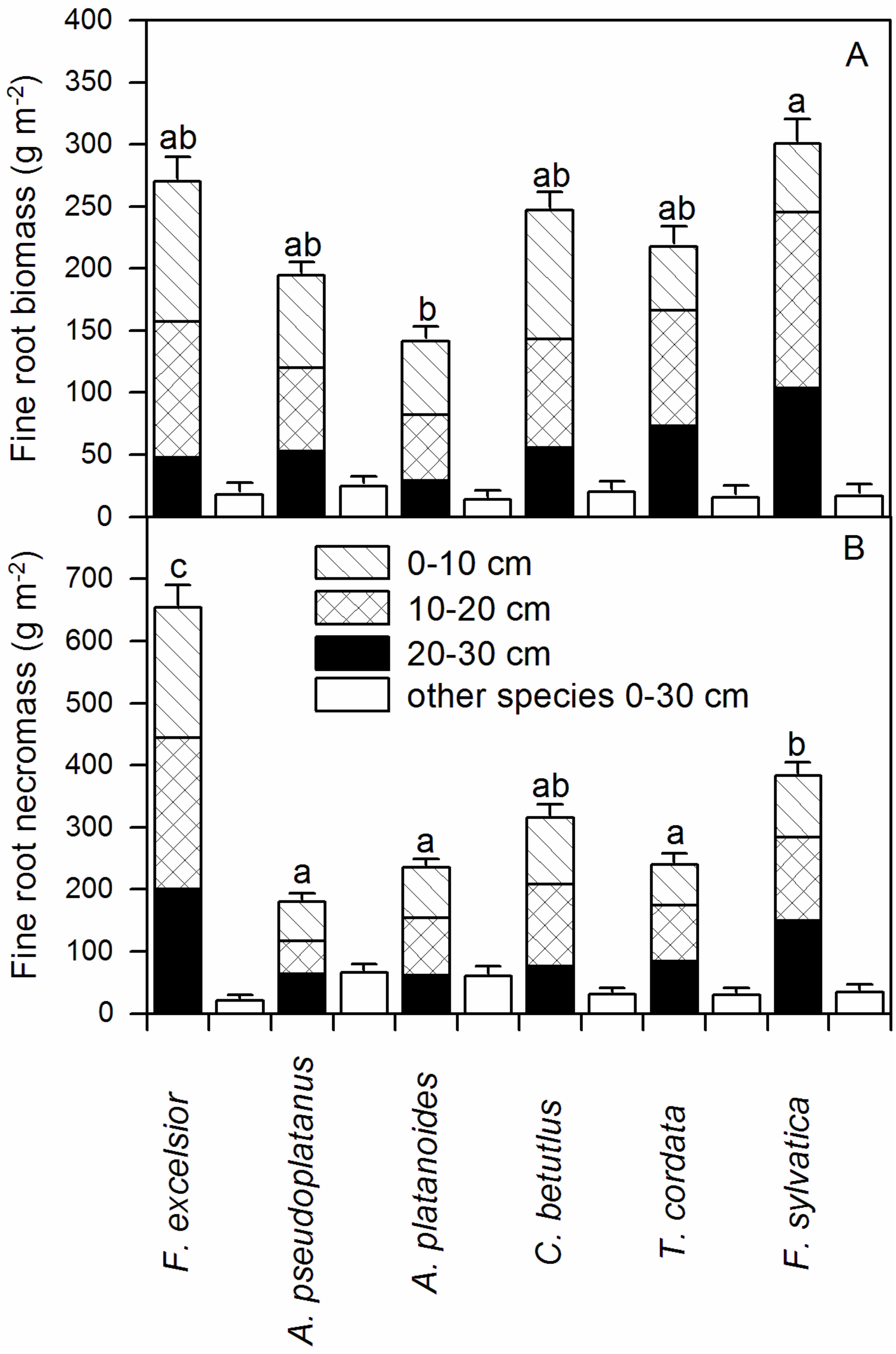

Supplement: Supplementary file 3 [file Image2.PDF]
